# National Survey of Pharmacist Awareness, Interest, and Readiness for Over-the-Counter Hearing Aids

**DOI:** 10.3390/pharmacy10060150

**Published:** 2022-11-12

**Authors:** Elizabeth S. Midey, Alexis Gaggini, Elaine Mormer, Lucas A. Berenbrok

**Affiliations:** 1 Department of Pharmacy and Therapeutics, School of Pharmacy, University of Pittsburgh, Pittsburgh, PA 15261, USA; 2 Department of Communication Science and Disorders, School of Health and Rehabilitation Sciences, University of Pittsburgh, Pittsburgh, PA 15213, USA

**Keywords:** over-the-counter hearing aids, hearing loss, community pharmacy, public health

## Abstract

Hearing loss is a major public health concern, affecting over 30 million Americans. Few adults who could benefit from hearing aids use them. Hearing aids are now available over-the-counter (OTC) for persons with perceived mild-to-moderate hearing loss. Community pharmacies will sell OTC hearing aids to increase public access to hearing healthcare. The purpose of this study was to describe pharmacist awareness, interest, and readiness to offer OTC hearing aids at community pharmacies. A multiple-item online survey was designed using the Theory of Planned Behavior and responses were collected from licensed pharmacists from July 2021 to December 2021. Descriptive statistics were used to summarize the 97 responses collected. Most respondents were not aware of the upcoming OTC hearing aid availability. Most respondents were somewhat or very interested in increasing their knowledge on OTC hearing aids, selling OTC hearing aids, and assisting patients with OTC hearing aid selection. Most respondents disagreed or strongly disagreed that they had the necessary knowledge to counsel patients on OTC hearing aids. The most reported supporting factor was training and educational resources. OTC hearing aids are a unique public health initiative which will expand patient access to hearing health care to community pharmacies.

## 1. Introduction

Hearing loss is a major public health concern. According to data from the National Institutes of Health (NIH), 30 million Americans have hearing loss in both ears, and approximately 28.8 million adults in the United States could benefit from hearing aids [1]. Despite these statistics, fewer than 30% of adults over age 70 who could benefit from hearing aids use them [2]. When left untreated, hearing loss is associated with increased risk of depression, social isolation, falls, and dementia [3,4,5,6]. Systematic reviews indicate that hearing aids can reduce the impairment and activity limitations caused by hearing loss [7]. For these reasons, the National Institute on Deafness and Other Communication Disorders (NIDCD) has prioritized solutions to improving hearing health care to meet this urgent public health problem [8]. Offering hearing healthcare in community pharmacies is an opportunity to address this concern given that nearly 90% of Americans live within 5 miles of a pharmacy [9].

The Reauthorization Act of 2017 required the U.S. Food and Drug Administration (FDA) to create and regulate a category of over-the-counter (OTC) hearing aids for adults with perceived mild-to-moderate hearing loss. The FDA issued its final rule establishing OTC hearing aids as an approved product category on 17 August 2022 [10]. The final rule was issued concurrently with regulatory requirements that define hearing aids as a medical device regulated by the FDA, distinct from personal sound amplification devices (PSAPs) which are not regulated as medical devices. These new standards are intended to improve the accessibility and affordability of hearing aids.

Because OTC hearing aids will not require a prescription or a medical evaluation, it is likely that community pharmacists will be called upon to support safe and effective use for patients contemplating the purchase of OTC hearing aids at the community pharmacy [11]. This support may include recognizing the signs and symptoms of hearing loss, assessing the individual’s need for referral, and managing patient’s expectations of hearing devices after purchase [12]. However, OTC hearing aids will be self-managed devices, indicating that pharmacists will not be expected to conduct hearing tests or device fittings.

The purpose of this study is to describe pharmacist awareness, interest, and readiness to offer OTC hearing aids at community pharmacies. Understanding pharmacist awareness, interest, and readiness for OTC hearing aids serves as a first step towards maximizing pharmacists’ ability to provide safe and effective supportive care to patients seeking hearing self-care at the community pharmacy. 

## 2. Materials and Methods

A 28-item online survey was designed to elicit responses regarding pharmacist awareness, interest, and readiness for OTC hearing aids. The survey was administered using Qualtrics (Provo, UT) and distributed through social media and an electronic newsletter distributed by a nationwide pharmacy organization representing community pharmacists. Survey questions probed pharmacists’ knowledge and interest in OTC hearing aids. Questions regarding barriers and facilitating factors were included to assess readiness. The Theory of Planned Behavior and the research framework of Dissemination and Implementation Science were used to develop survey questions [13]. Descriptive statistics were used to summarize participant demographics, knowledge and perspectives, and the constructs of awareness, interest, and readiness. We further assessed the differences in pharmacist awareness, interest, and readiness across employer or business type, practice sites, and personal hearing device use. The University of Pittsburgh Institutional Review Board approved this cross-sectional descriptive study.

Inclusionary criteria included U.S. pharmacists over 21 years of age licensed to practice. Participation was anonymous and voluntary. No compensation or incentives were provided to participants. Participants were able to stop taking the survey at any time. Prior to national distribution, the survey was piloted using a convenience sample of practicing pharmacists known to the investigators to gather feedback on survey content and design. The pilot sample was recruited through email, text messaging, and use of instant messaging on social media platforms. The pilot survey included an optional free response prompt at the conclusion of each section to elicit user feedback on survey content and navigation. Responses from the pilot were used to revise the survey structure and content in preparation for survey distribution and data collection.

The finalized survey was opened in July 2021 and responses were accepted until December 2021, after which the survey link was closed. The survey was initially distributed through a three-day advertisement in a daily email newsletter published by a national community pharmacy organization, and a second advertisement was distributed prior to the end of the recruitment period. Participants were also recruited through social media platforms, including LinkedIn, Facebook, and Reddit to broadly capture perspectives of practicing pharmacists. The survey was shared on the investigators’ personal pages in addition to interest groups, pages, and forums for pharmacists on the various social media platforms. Pharmacist interest groups based in specific U.S. states were targeted to ensure the study population included participants from the Northeast, Midwest, South, and West regions of the U.S.

The survey instrument collected participant age, professional experience, practice setting, and personal use of hearing devices. Additional survey questions were included to assess participant awareness of the legal status and availability of OTC hearing aids, and perceptions of the patient population served by the participant. Interests were probed through items focusing on knowledge, sale of, and resources for OTC hearing aids. To assess readiness, participant perceptions of competence, barriers, and facilitating factors to offering OTC hearing aids in a community pharmacy were also collected. Questions also explored pharmacist and employer preparation to sell OTC hearing aids, current sale of unregulated hearing devices like personal sound amplification products, and access to education and training resources during hours of employment. Lastly, participants were asked to share their concerns and perceived benefits of providing OTC hearing aids in the pharmacy. The full survey instrument is included as Appendix A.

## 3. Results

### 3.1. Respondents

The survey received 97 total responses. Due to the dissemination methods, the response rate could not be determined. Eligible respondents held practice sites in 27 states, representing the Northeast, Midwest, South, and West regions of the United States [14]. Respondent demographics are summarized in Table 1.

### 3.2. Awareness

Of 88 respondents, 57 (64.77%) were not aware of pending OTC hearing aid availability prior to the survey. Most respondents, 48 (54.55%), stated that they were “not at all familiar” with OTC hearing aids prior to the survey. Of 87 respondents, 68.97% reported frequent interaction with persons with hearing loss who use hearing devices, while 50.57% of respondents reported interacting frequently with persons with hearing loss who do not use hearing devices. Additional data about awareness is summarized in Table 2.

Fifty (67.6%) of the respondents who had never personally used a hearing device were not aware that OTC hearing aids will be available in pharmacies prior to taking the survey. Among the respondents who had used a professionally fitted hearing aid, 6 (60%) were not aware. Of the respondents who had used a personal sound amplification product, only 2 (28.6%) were not aware of OTC hearing aids.

### 3.3. Interest

Of 85 respondents, 94.1% were somewhat or very interested in increasing their knowledge about OTC hearing aids. Preferred education resources for OTC hearing aids from 79 respondents included continuing education (91.1%), followed by webinars (59.5%), and certificates or micro-credentials (41.8%). The majority of 85 respondents were somewhat or very interested in selling OTC hearing aids at their own practice (71.8%) and assisting patients with OTC hearing aid selection (72.9%). Pharmacist interest data is summarized in Table 3. 

Pharmacists employed by a mass merchant, independent pharmacy, or a traditional chain were most interested in selling OTC hearing aids at their practice. All 7 (100%) of the mass merchant pharmacists responding to “How interested are you in selling OTC hearing aids in your practice?” indicated that they were somewhat or very interested. Of the 39 independent pharmacists who responded, 34 (87.2%) were interested, and 11 (78.6%) of 14 traditional chain pharmacists were interested. Of the five respondents who stated that they were not interested in increasing their knowledge about OTC hearing aids, all (100%) stated that they were not interested in selling OTC hearing aids at their own practice and not interested in assisting patients with OTC hearing aid selection.

Respondents practicing in rural, suburban, and urban pharmacies (*n* = 71) reported differing levels of interest in selling OTC hearing aids. Out of 23 respondents in rural locations, 82.61% were somewhat or very interested in selling OTC hearing aids, followed by 82.35% of pharmacists in urban settings (*n* = 17). Of 31 respondents in suburban locations, 77.42% indicated that they were somewhat or very interested in selling OTC hearing aids. Of 17 respondents at urban sites, 58.82% agreed or strongly agreed that patients in their community would purchase OTC hearing aids, followed by 74.19% (*n* = 23) of 31 respondenents from suburban locations. Of 23 respondents at rural sites, 56.52% (*n* = 13) agreed and 21.74% (*n* = 5) strongly agreed that patients would purchase OTC hearing aids.

Of 7 respondents who had personally used a PSAP, 6 (85.7%) were very interested in gaining more knowledge about OTC hearing aids and 1 respondent (14.3%) was somewhat interested. All (100%) of 9 respondents who had used a professionally fitted hearing aid were either somewhat (*n* = 3) or very interested (*n* = 6) in increasing their knowledge. Of 72 respondents who had never used a hearing device, only 5 (6.94%) remained not interested in increasing their knowledge of OTC hearing aids. Additionally, 6 of 7 (85.7%) of the respondents who had used a PSAP were very interested in selling OTC hearing aids in their practice, compared to 4 of 9 (44.4%) of respondents who used a professionally fitted hearing aid and 16 of 72 (22.2%) of respondents who had never used a hearing device.

### 3.4. Readiness

Of 85 respondents, 59 (69.41%) agreed or strongly agreed that patients in their community would purchase OTC hearing aids at a community pharmacy. However, 57 (67.06%) of respondents disagreed or strongly disagreed that they had the necessary knowledge to counsel patients on OTC hearing aids. Of 82 respondents, 70 (85.37%) considered training and educational resources for pharmacy staff to be a supporting factor for integrating OTC hearing aids into practice, followed by professional connections to hearing healthcare professionals like audiologists 51 (62.2%) and expertise of pharmacy colleagues 49 (59.76%). The most common barriers to selling OTC hearing aids expected were pharmacist time and workflow constraints 76 (92.68%), available training and/or continuing education for pharmacists 54 (65.85%), and level of pharmacist expertise 54 (65.85%). Additional readiness data is summarized in Table 4.

Most respondents employed at traditional chains (76.92%), mass merchants (85.71%), and supermarkets (60.0%) endorsed that the decision to sell OTC hearing aids at their pharmacy was not within their control. The remainder of respondents in these categories stated they were unsure if they would offer OTC hearing aids. Respondents employed at independent pharmacies (*n* = 39) were the only category of respondents stating they intended to offer OTC hearing aids once available, 30.8% (*n* = 12). Additionally, 43.59% of 39 respondents at independent pharmacies stated that their workplace would allow time for education and training about OTC hearing aids during work hours. In contrast, only 1 (7.69%) traditional chain, 2 (2.86%) mass merchant, and 0 (0.00%) of supermarket pharmacists believed that their employer would allow time for education and training during work hours. Employer readiness is summarized in Table 5. 

## 4. Discussion

OTC hearing aids represent a unique opportunity for community pharmacists to advance public health by increasing patient access to hearing healthcare. Prior to this study, little was known about pharmacist awareness, interest, and readiness to take on this new public health initiative intended to increase accessibility and affordability of hearing aids to the millions of Americans with hearing loss. To our knowledge, this is the first nationwide survey of pharmacists intended to capture pharmacist perceptions of OTC hearing aids and their intention and readiness to participate in hearing health care.

Pharmacists surveyed in this study in general lacked awareness and readiness for OTC hearing aids. Although most respondents were not aware of upcoming OTC hearing aid availability, a majority reported frequent interactions with patients who have hearing loss and use hearing devices. This discordance may further illustrate a lack of interest and engagement in hearing loss by pharmacists. Following the FDA’s final ruling on OTC hearing aids, published 17 August 2022, more pharmacists and patients are likely to be aware that OTC hearing aids will be available for purchase at locations like community pharmacies. As awareness among the public increases, pharmacists and their employers should ready themselves for the sale of these products to persons with hearing loss.

Most respondents also expressed interest in learning more about OTC hearing aids, assisting patients with OTC hearing aid selection, and selling OTC hearing aids at their practice site. Pharmacist respondents who had used PSAPs indicated higher levels of interest in comparison to respondents who had never used a hearing device or who had used professionally fitted hearing aids. This may be due to the fact that PSAPs have long been available to consumers at community pharmacies without the need for a prescription or professional services. Unlike OTC hearing aids, PSAPs are not regulated by the FDA as medical devices and are not intended to treat hearing loss [11]. Respondents practicing at independent pharmacies, mass merchant pharmacies, and traditional chains had the highest levels of overall interest in selling OTC hearing aids which may indicate where OTC hearing aids will likely be sold. These pharmacy location types have experience offering medical devices to their patients including durable medical equipment, whereas supermarket pharmacies may not. Interest levels for selling OTC hearing aids were also highest among pharmacists practicing in rural and urban settings which have been found to be underserved by audiologists in a previous analysis [15]. Notably a majority of respondents at rural practice sites strongly agreed that patients would purchase OTC hearing aids from a community pharmacy. This may be due to the fact that persons in rural areas have little access to audiology services [15]. Because community pharmacies are highly accessible health care locations for the overall U.S. population, audiologists may consider establishing collaborative working relationships with pharmacists to better serve persons with hearing loss in rural areas [9,16]. 

There was discordance across respondents regarding the ability to offer OTC hearing aids. Most respondents at traditional chain, mass merchants, and supermarket pharmacies stated that whether or not they offer OTC hearing aids in their practice was not within their control. The remainder were unsure. Respondents at independent pharmacies were the only category that indicated an intent to offer OTC hearing aids at their practice, mostly likely due to the nature and individual control of independent ownership. Respondents at independent pharmacies frequently stated that their workplace would offer time for education and training on OTC hearing aids. This may indicate that independent pharmacies will prepare their pharmacists for OTC hearing aids by offering additional education and training. 

It is relevant to consider existing literature describing pharmacist knowledge and perspectives on adopting new patient care services in pharmacy practice. A recent survey study by Nichols et al. characterized the knowledge and perspectives of community pharmacy preceptors regarding cannabidiol (CBD) approximately two years after the federal reclassification of CBD. The authors reported that more respondents were uncomfortable than comfortable counseling on CBD products, similar to our findings in the context of OTC hearing aids [17]. A second study by Smith and Rains surveyed pharmacists in Arkansas to investigate their preparedness to implement point-of-care testing services in community pharmacies [18]. A majority (52.2%) of 25 respondents in the POCT study requested additional resources beyond an initial training program. Because the majority of respondents in our study did not believe they had the necessary knowledge to counsel patients on OTC hearing aids, there exists an opportunity to provide pharmacists with additional training and continuing education resources to fill this gap. Correspondingly, a high percentage of respondents indicated that they were interested in increasing their knowledge on OTC hearing aids, and most pharmacists were interested in continuing education. However, our data suggests that extensive education and training may not be not needed to ready pharmacists for OTC hearing aids. Nearly 80% of respondents indicated that they would feel prepared to assist patients with OTC hearing aids within 6 months. Prior to this study, the University of Pittsburgh created a 2.5 h continuing pharmacy education course on OTC hearing aids which is available online for pharmacists [19].

This study is limited by the utilization of a convenience sample and by the timing of data collection. Firstly, due to convenience, the respondent sample may not comprehensively represent pharmacist awareness, interest, and readiness for OTC hearing aids despite responses from all four regions of the U.S. and a diverse profile of employers. It is also possible that pharmacists with interest in OTC hearing aids self-selected to provide responses and, as such, are overrepresented in our study. Secondly, in October 2021 the FDA released proposed OTC hearing aid regulations increasing public awareness to OTC hearing aids as a new category of hearing devices. Because regulations were proposed in the middle of our collection period (July 2021–December 2021), pharmacists who responded after may have answered differently than how they would have responded before the regulations were proposed.

## 5. Conclusions

OTC hearing aids are a unique public health initiative which will expand patient access to hearing health care to community pharmacies. Pharmacist awareness of OTC hearing aids was limited at the time of this study. Pharmacists were interested in learning more about OTC hearing aids and in assisting patients with the selection of these devices. Most pharmacists did not feel prepared to counsel patients on OTC hearing aid use. The development and dissemination of training and educational resources for pharmacists would be beneficial to the success of OTC hearing aids as a public health initiative.

## Figures and Tables

**Table 1:** Demographics of Respondents.

Characteristic	Category	Respondents (% *)
Age (*n* = 97)	21–29	10 (10.3)
	30–39	32 (33.0)
	40–49	18 (18.6)
	50–59	18 (18.6)
	60–69	16 (16.5)
	70–79	3 (3.0)
Personal use of hearing devices ^a^ (*n* = 97)	Never used a hearing device	81 (79.4)
	Hearing aid professional fitted by an audiologist	10 (9.8)
	Personal sound amplification product	7 (6.9)
	Other	4 (3.9)
Current practice as community pharmacist (*n* = 94)	Yes	77 (81.9)
	No	17 (18.0)
Primary practice setting ^b,c^ (*n* = 77)	Suburban	35 (45.5)
	Rural	25 (32.5)
	Urban	17 (22.0)
Employer or business (*n* = 90)	Independent	41 (45.6)
	Traditional chain	15 (16.7)
	Mass merchant	7 (7.8)
	Outpatient	6 (6.7)
	Supermarket	5 (5.6)
	Other	16 (17.8)
Primary U.S. state of practice ^d^ (*n* = 90)	South	41 (45.6)
	Midwest	25 (27.8)
	Northeast	16 (17.8)
	West	8 (8.9)

* Totals may not add to 100% due to rounding. ^a^ Participants could select all that applied. ^b^ Based on self-report. ^c^ Question only presented to respondents currently practicing as a community pharmacist (*n* = 77). ^d^ Based on U.S. Census Bureau regions and divisions of the United States [14].

**Table 2:** Awareness of Hearing Health.

Question	Category	Respondents (% *)
Awareness of new OTC hearing aid category prior to survey (*n* = 88)	Yes	31 (35.2)
	No	57 (64.8)
Familiarity with OTC hearing aids (*n* = 88)	Not at all familiar	48 (54.5)
	Somewhat familiar	38 (43.2)
	Very familiar	2 (2.3)
Frequency of interaction with persons with hearing loss who do not use hearing devices ^a^ (*n* = 87)	Never	3 (3.5)
	Rarely	5 (5.8)
	Occasionally	26 (29.9)
	Frequently	44 (50.6)
	Unknown	9 (10.3)
Frequency of pharmacist interaction with persons with hearing loss who use hearing devices ^a^ (*n* = 87)	Never	4 (4.6)
	Rarely	6 (6.9)
	Occasionally	12 (13.8)
	Frequently	60 (69.0)
	Unknown	5 (5.8)

* Totals may not add to 100% due to rounding. ^a^ Respondent perception of patient population, not prevalence.

**Table 3:** Interest in OTC Hearing Aids.

Question	Category	Respondents (% *)
Interest in increasing knowledge on OTC hearing aids (*n* = 85)	Not interested	5 (5.9)
	Somewhat interested	31 (36.5)
	Very interested	49 (57.7)
Interest in resources to learn about OTC hearing aids ^a,b^ (*n* = 79)	Continuing education	72 (91.1)
	Webinars	47 (59.5)
	Certificates/micro-credentials	33 (41.8)
	On-the-job training	32 (40.5)
	Informal personal research	21 (26.6)
	Conferences	19 (24.0)
Interest in selling OTC hearing aids at own practice (*n* = 85)	Not interested	24 (28.2)
	Somewhat interested	38 (44.7)
	Very interested	23 (27.0)
Interest in assisting patients with OTC hearing aid selection (*n* = 85)	Not interested	23 (27.1)
	Somewhat interested	37 (43.5)
	Very interested	25 (29.4)

* Totals may not add to 100% due to rounding. ^a^ Respondents selected all choices that applied. Response was not required. ^b^ Question only presented to respondents somewhat or very interested in increasing their knowledge (*n* = 80).

**Table 4:** Readiness to Offer OTC Hearing Aids.

Question	Category	Respondents (% *)
I have the necessary knowledge to counsel patients on OTC hearing aids (*n* = 85)	Strongly disagree	37 (43.5)
	Disagree	20 (23.5)
	Neutral	21 (24.7)
	Agree	5 (5.9)
	Strongly agree	2 (2.4)
Patients in my community would purchase OTC hearing aids at a community pharmacy for their personal use (*n* = 85)	Strongly disagree	4 (4.7)
	Disagree	3 (3.5)
	Neutral	19 (22.4)
	Agree	37 (43.5)
	Strongly agree	22 (25.9)
Expected supporting factors as OTC hearing aids become available ^a^ (*n* = 82)	Training and educational resources for pharmacy staff	70 (85.4)
	Professional connections to hearing health care professionals like audiologists	51 (62.2)
	Expertise of pharmacy colleagues	49 (59.8)
	Support from place of employment	44 (53.7)
	Supportive leadership	28 (34.2)
	Culture or mission of place of employment	23 (28.1)
	Affiliation with research institutions	10 (12.2)
	Other	2 (2.4)
Expected barriers as OTC hearing aids become available ^a^ (*n* = 82)	Pharmacist time and workflow constraints	76 (92.7)
	Available training and/or continuing education for pharmacists	54 (65.9)
	Level of pharmacist expertise	54 (65.9)
	Staff resources	48 (58.5)
	Patient understanding of appropriate OTC hearing aid use	34 (41.5)
	Support from place of employment	34 (41.5)
	Patient interest in use of OTC hearing aids	20 (24.4)
	Other	2 (2.4)
When prepared to assist patients with OTC hearing aids, if available today (*n* = 81)	Within 30 days	36 (44.4)
	Within 6 months	28 (34.6)
	Within 1 year	1 (1.2)
	After 1 or more years	0 (0.0)
	I do not intend to offer OTC hearing aids at my pharmacy	9 (11.1)
	Not applicable	7 (8.6)
Benefits to offering OTC hearing aids at the pharmacy ^a^ (*n* = 81)	Improved patient access to hearing aids	71 (87.7)
	Flexible treatment options	61 (75.3)
	Increased patient interest in the use of hearing aids	42 (51.9)
	Increased revenue at my place of employment	35 (43.2)
	Reduction of health risks associated with hearing loss	33 (40.7)
	Other	1 (1.2)
	None	4 (4.9)
Concerns about offering OTC hearing aids at the pharmacy ^a^ (*n* = 81)	Improper or unsafe use by patients	54 (66.7)
	Increased personal or professional liability	37 (45.7)
	Quality of research on OTC hearing aid safety and effectiveness	32 (39.5)
	Lack of patient trust in new technology	20 (24.7)
	Other	5 (6.2)
	None	9 (11.1)

* Totals may not add to 100% due to rounding. ^a^ Respondents selected all choices that applied.

**Table 5:** Employer Readiness to Offer OTC hearing Aids in the Pharmacy Setting.

Characteristic	Value	Respondents (%)
Current participant or employer preparing to offer OTC hearing aids (*n* = 82)	Yes	3 (3.7)
	No	20 (24.4)
	Unsure	12 (14.6)
	Not applicable	47 (57.3)
Current sale of assistive hearing devices at workplace (*n* = 83)	Yes	4 (4.9)
	No	56 (68.3)
	Unsure	14 (17.0)
	Not applicable	8 (9.8)
Workplace allowance of education and training time for OTC hearing aids (*n* = 82)	Yes	24 (29.3)
	No	16 (19.5)
	Unsure	35 (42.7)
	Not applicable	7 (8.5)
Intent to offer OTC hearing aids as a service when available (*n* = 81)	Yes	12 (14.8)
	No	3 (3.7)
	Unsure	20 (24.7)
	Not in my control	37 (45.7)
	Not applicable	9 (11.1)

## Data Availability

The data presented in this study are available on request from the corresponding author.

## Appendix A

Pharmacist Awareness, Interest, and Readiness Regarding OTC Hearing Aids (NCPA July 2021)

Start of Block: Default Question Block

- Q12 The purpose of this research study is to assess the awareness, interest, and readiness of pharmacists regarding OTC hearing aids. For that reason, we will survey community pharmacists in the U.S. by asking them to complete a brief questionnaire that will take approximately 10 min. All participants must be 21 years of age or older. If you are willing to participate, our questionnaire will ask about your background (e.g., age, years practicing as a pharmacist, practice setting and location), as well as about your knowledge and views regarding OTC hearing aids. There are minimal foreseeable risks associated with this project (e.g., discomfort answering survey questions.) There are no direct benefits to you. This is an anonymous questionnaire, and your responses will not be identifiable in any way. All responses are confidential, and results will be stored electronically in password-protected files. Your participation is voluntary, and you may stop completing the survey at any time. This study is being conducted by Lucas Berenbrok (berenbrok@pitt.edu) and Elizabeth Midey (esm53@pitt.edu) at the University of Pittsburgh School of Pharmacy, who can be reached by email if you have any questions.
- Q2 What is your age?

▼ Under 21 (2299) … 90+ (2369)

Skip To: End of Survey If What is your age? = Under 21

- Q65 Have you ever personally used any of the following hearing devices? (Select all that apply)
  ○ Hearing aid professionally fitted by an audiologist (1)
  ○ Personal sound amplification product (PSAP) (4)
  ○ I have never used a hearing device (2)
  ○ Other (please specify) (3) _______________________________________________

- Q29 Do you currently practice as a licensed community pharmacist in any capacity? (e.g., full-time, part-time, per diem, on-call)
  ○ Yes (1)
  ○ No (2)

Display This Question:

If Do you currently practice as a licensed community pharmacist in any capacity? (e.g., full-time, p... = Yes)

- Q62 For how many years have you practiced as a licensed pharmacist in a community pharmacy? (Please round up to the nearest year)

▼ 1 (203) … 50+ (252)

Display This Question:

If Do you currently practice as a licensed community pharmacist in any capacity? (e.g., full-time, p... = Yes

- Q78 Which of the following best describes your primary practice location?
  ○ Urban (3)
  ○ Suburban (2)
  ○ Rural (1)

- Q60 Which of the following best describes your employer or your business.
  ○ Traditional chain (e.g., CVS, Walgreens, Rite Aid) (1)
  ○ Mass merchant (e.g., Costco, Walmart, Sam’s Club) (2)
  ○ Independent (3)
  ○ Supermarket (e.g., ACME, Kroger) (4)
  ○ Outpatient (e.g., healthcare system) (5)
  ○ Other (please specify) (8) ______________________________________________

- Q59 Where is your primary location of work? (i.e., the location where you work for the majority of the time)

▼ Alabama (9) … Wyoming (58)

- Q6 Before taking this survey, were you aware that patients will soon be able to purchase hearing aids over-the-counter? i.e., without medical evaluation from a doctor or a fitting by an audiologist
  ○ Yes (1)
  ○ No (2)

Display This Question:

If Before today, were you aware that patients will be able to purchase hearing aids over-the-counter in = Yes

- Q8 If yes, where have you heard about OTC hearing aids before? (Select all that apply)
  ○ Colleagues (1)
  ○ Continuing education (2)
  ○ News outlets (7)
  ○ Professional organization (4)
  ○ Social media (5)
  ○ Other (please specify) (6) ______________________________________________

- Q9 Before taking this survey, how familiar were you with OTC hearing aids?
  ○ Not at all familiar (this is the first time I have heard about OTC hearing aids) (1)
  ○ Somewhat familiar (I have heard about OTC hearing aids, but I have no knowledge of the law or regulations) (2)
  ○ Very familiar (I am actively awaiting FDA regulations so that I can offer OTC hearing aids in my pharmacy) (3)

- Q77 How often, at work, do you interact with the following persons?

	Never	Rarely (once per year or less)	Occasionally (several times per year)	Frequently (daily to monthly)	Unknown
Persons with hearing loss who DO NOT use hearing devices					
Persons with hearing loss who USE hearing devices					
Persons who are deaf					

- Q47 How confident are you in communicating with the following persons in a healthcare setting?

	Not at all confident	Slightly confident	Somewhat confident	Quite confident	Extremely confident
Persons without hearing loss					
Persons with partial hearing loss					
Persons who are deaf					

- Q10 How interested are you in increasing your knowledge about OTC hearing aids?
  ○ Not interested (1)
  ○ Somewhat interested (2)
  ○ Very interested (3)

Display This Question:

If How interested are you in increasing your knowledge about OTC hearing aids? != Not interested

- Q63 What resources would you be interested in using to learn more about OTC hearing aids? (Select all that apply)
  ○ Certificates/micro-credentials (1)
  ○ Conferences (2)
  ○ Continuing education (3)
  ○ Informal personal research (4)
  ○ On-the-job training (5)
  ○ Webinars (7)
  ○ Other (please specify) (8) _____________________________________________
  ○ None of the above (9)

- Q11 How interested are you in selling OTC hearing aids in your practice?
  ○ Not interested (1)
  ○ Somewhat interested (2)
  ○ Very interested (3)

- Q17 How interested are you in assisting patients with the selection of OTC hearing aids?
  ○ Not interested (1)
  ○ Somewhat interested (2)
  ○ Very interested (3)

- Q79 Please select your level of agreement with each statement below.

- Q70 I believe that I have the necessary knowledge to counsel patients on OTC hearing aids.
  ○ Strongly disagree (1)
  ○ Disagree (2)
  ○ Neutral (3)
  ○ Agree (4)
  ○ Strongly agree (5)

- Q69 I believe that patients in my community would purchase OTC hearing aids at a community pharmacy for their personal use.
  ○ Strongly disagree (1)
  ○ Disagree (2)
  ○ Neutral (3)
  ○ Agree (4)
  ○ Strongly Agree (5)

- Q24 What factors would support the integration of OTC hearing aids into pharmacy practice? (Select all that apply)
  ○ Affiliations with research institutions (4)
  ○ Professional connections to hearing healthcare professionals like audiologists (1)
  ○ Culture or mission of place of employment (6)
  ○ Expertise of pharmacy colleagues (9)
  ○ Support from place of employment (3)
  ○ Supportive leadership (5)
  ○ Training and educational resources for pharmacy staff (11)
  ○ Other (please specify): (8) ______________________________________________

- Q21 What barriers do you expect pharmacists to face as OTC hearing aids become available at the pharmacy? (Select all that apply)
  ○ Available training and/or continuing education for pharmacists (1)
  ○ Pharmacist time and workflow constraints (2)
  ○ Level of pharmacist expertise (3)
  ○ Patient interest in use of OTC hearing aids (4)
  ○ Patient understanding of appropriate OTC hearing aid use (5)
  ○ Staff resources (6)
  ○ Support from place of employment (7)
  ○ Other (please specify): (9) ______________________________________________

- Q32 Are you or your employer preparing to offer OTC hearing aids at your pharmacy?
  ○ Yes (1)
  ○ No (2)
  ○ Unsure (5)
  ○ Not applicable (3)

Display This Question:

If Are you or your employer preparing to offer OTC hearing aids at your pharmacy? = Yes

- Q35 If yes, what resources have you used to prepare? (Select all that apply)
  ○ Conferences (5)
  ○ Continuing education (6)
  ○ Informal personal research (7)
  ○ On-the-job training (8)
  ○ Webinars (10)
  ○ Other (please specify) (11) _____________________________________________

- Q64 Does your place of work currently sell any assistive hearing devices such as hearing aids, personal sound amplification products (PSAPs), assisted listening devices, or hearables?
  ○ Yes (please specify) (1) ________________________________________________
  ○ No (2)
  ○ Unsure (3)
  ○ Not applicable (5)

- Q19 Will your place of work allow time for your education and training regarding OTC hearing aids during work hours?
  ○ Yes (1)
  ○ No (3)
  ○ Unsure (2)
  ○ Not applicable (4)

- Q37 Do you intend to offer OTC hearing aids as a service in your pharmacy once these products are available?
  ○ Yes (1)
  ○ No (2)
  ○ Unsure (3)
  ○ Not in my control (5)
  ○ Not applicable (4)

- Q36 If OTC hearing aids were available today, when would you feel prepared to assist patients with these devices?
  ○ Within 30 days (1)
  ○ Within 6 months (2)
  ○ Within 1 year (3)
  ○ After 1 or more years (4)
  ○ I do not intend to offer OTC hearing aids at my pharmacy (7)
  ○ Not applicable (8)

- Q39 What benefits do you see to offering OTC hearing aids at the pharmacy? (Select all that apply)
  ○ Flexible treatment options for persons with hearing loss (7)
  ○ Improved patient access to hearing aids (2)
  ○ Increased patient interest in the use of hearing aids (1)
  ○ Increased revenue at my place of employment (3)
  ○ Reduction of health risks associated with hearing loss (4)
  ○ Other (5) __________________________________________________
  ○ None (6)

- Q40 What concerns do you have about offering OTC hearing aids at the pharmacy? (Select all that apply)
  ○ Improper or unsafe use by patients (1)
  ○ Lack of patient trust in new technology (2)
  ○ Increased personal or professional liability (7)
  ○ Quality of research on OTC hearing aid safety and effectiveness (3)
  ○ Other (5) __________________________________________________
  ○ None (6)

- Q54 Please share any additional comments regarding OTC hearing aids.
  ________________________________________________________________
  ________________________________________________________________
  ________________________________________________________________
  ________________________________________________________________
  ________________________________________________________________

End of Block: Default Question Block
